# Type 2 diabetes prevention programs at the community and municipal level: a scoping review of controlled intervention studies

**DOI:** 10.1186/s12889-026-28319-8

**Published:** 2026-07-01

**Authors:** Dominique Michels, Carolin Walter, Alana Grathwohl-Karl, Johanna Pfau, Hannah Haumann, Stefanie Joos, Daniela Fröhlich

**Affiliations:** 1 Institute of General Practice and Interprofessional Care, University Hospital Tuebingen, Tuebingen, 72076 Germany; 2 Department of Population-Based Medicine, Institute of Health Sciences, University Hospital Tuebingen, Tuebingen, 72076 Germany

**Keywords:** Diabetes, Prevention, Evidence-based approaches, Health promotion, Public health service

## Abstract

**Background:**

Type 2 diabetes mellitus poses a major challenge to public health globally due to its rising prevalence and high proportion of patients who are undiagnosed, untreated and/or uncontrolled. Lifestyle interventions targeting nutrition, physical activity, and further health-related behaviors such as weight and stress management can prevent the onset of type 2 diabetes mellitus or delay progression from prediabetes to manifest disease. Although lifestyle interventions are widely used in clinical trials and national programs, there is limited evidence on how such interventions are designed, adapted, and reported in community, municipal, and public health settings.

**Methods:**

A scoping review was conducted using the Population Concept Context framework. Systematic searches were performed in PubMed, Web of Science Core Collection, CINAHL, and CENTRAL. The CDSR was used for citation tracking to identify relevant primary studies. Additional searches included grey literature, organizational websites, and an AI-assisted search (undermind.ai). Searches covered studies published in English or German between January 2014 and May 2025 and were conducted on 28 May 2025. Eligible studies examined lifestyle-based interventions targeting physical activity, nutrition, or broader behavioral change among healthy adults and adults at increased risk of developing type 2 diabetes, including individuals with prediabetes or metabolic syndrome, delivered in community, municipal, or public health settings. Inclusion was restricted to controlled study designs ((cluster-)randomized controlled trials, non-randomized controlled studies, or controlled clinical trials). Feasibility studies, pilot studies, uncontrolled implementation studies, and process evaluations were excluded. Pharmacological interventions were not considered. Data were charted and synthesized descriptively.

**Results:**

Of 464 records identified, 26 studies were included. Interventions were largely adapted from established prevention programs and delivered through group-based education, with limited reporting of theoretical frameworks. Definitions and operationalization of “community-based” delivery varied across studies, and intervention duration and maintenance components were heterogeneous. The evidence base was dominated by studies conducted in high-income countries, with limited representation from lower-resource settings.

**Conclusions:**

This scoping review highlights the need for greater representation of studies from lower-resource settings and theoretically grounded and methodologically rigorous prevention approaches in the community setting. The potential of community-based initiatives offers valuable guidance for further development and scaling future diabetes prevention efforts.

**Trial registration:**

Open Science Framework (osf.io/zafg5).

**Supplementary Information:**

The online version contains supplementary material available at 10.1186/s12889-026-28319-8.

## Background

### Rationale

Type 2 diabetes mellitus (T2DM) is one of the most significant global public health challenges. T2DM is characterized by its chronic progression, high comorbidity burden, and substantial economic costs. Current estimates indicate that 589 million adults were living with T2DM in 2024, with projections suggesting that this number could rise to 853 million by 2050 [1]. Approximately 43% of individuals with diabetes remain undiagnosed, particularly in low- and middle-income countries, where limited access to healthcare and structural inequities contribute to delayed detection [1]. Prediabetes, defined by elevated blood glucose markers such as impaired fasting glucose (IFG) or impaired glucose tolerance (IGT), represents a critical yet reversible stage in the progression toward T2DM [2].

Lifestyle-based interventions, including structured programs targeting nutrition, physical activity, and further behavioral and lifestyle changes like weight and stress management, can effectively delay or prevent the onset of T2DM among individuals at elevated metabolic risk [3–7]. In several countries, national initiatives have been launched over the past two decades to translate evidence-based approaches into practical diabetes prevention strategies [8–11]. Landmark trials such as the United States National Diabetes Prevention Program (NDPP) and the Finnish Diabetes Prevention Study (FIN-DPS) reported risk reductions of up to 58% among individuals with IGT or IGT + IFG [4, 12]. These effects exceeded those observed for pharmacological interventions such as metformin, which primarily delay, rather than prevent, disease onset [12]. National rollouts of such interventions in countries including Finland, the United States, and the United Kingdom (National Health Service Diabetes Prevention Programme, NHS DPP) have provided evidence of feasibility and effectiveness under real-world conditions, albeit showing smaller effects than in clinical trials [13–15]. At the same time, persistent challenges have been reported such as limited reach, low retention, as well as underrepresentation of young adults, ethnic minorities, and socioeconomically disadvantaged groups [10, 13]. In addition, structural and operational barriers, including constrained resources, heterogeneous populations, and difficulties integrating programs into existing healthcare infrastructures, further limit the scalability of traditional prevention models [10, 13, 16].

Against this backdrop, community-, municipal and public-health-based settings are receiving increased attention as promising avenues for expanding the reach and impact of diabetes prevention efforts. Embedded within local social, cultural, and organizational structures, these settings offer unique opportunities to tailor interventions to the needs of specific populations, enhance accessibility and cultural relevance, and strengthen long-term engagement. In this context, community, municipal, and public or primary health care settings refer to population-oriented environments for health promotion and disease prevention that are accessible and embedded within local communities. These include non-clinical venues such as community centers, and local (health) organizations, as well as community-linked health services such as public health departments, community health centers, and primary care practices that provide first-contact, accessible, and preventive services. This understanding aligns with World Health Organization (WHO) definitions emphasizing accessibility, community involvement, and population-level approaches to health promotion [17, 18]. Despite this potential, the evidence on diabetes prevention initiatives delivered in community and public health contexts remains fragmented [19, 20].

Existing reviews have largely focused on clinical interventions, large-scale national diabetes prevention programs (DPPs), or specific populations, with an emphasis on effectiveness and outcomes. In contrast, this review adopts a scoping approach to examine how diabetes prevention interventions are conceptualized, designed, adapted, implemented, and reported within community, municipal, and public health delivery structures. By focusing on intervention components, adaptation processes, and reporting practices, it addresses an underexplored dimension of implementation in population-based settings. This perspective complements clinically oriented and “real-world” DPP evaluations by providing a structured synthesis of how interventions are operationalized across diverse community and public health contexts [19, 20].

### Objectives

The present scoping review identifies and synthesizes diabetes prevention interventions that (a) target healthy individuals, individuals with prediabetes (defined by the WHO [21] and American Diabetes Association (ADA) [2]), or populations at increased risk for T2DM, including those with metabolic syndrome, (b) emphasize lifestyle or behavioral change, and (c) are implemented in public health or municipal or community settings. The inclusion of healthy individuals reflects a population-level prevention perspective, recognizing that early behavioral interventions may reduce the development of risk factors and contribute to lowering overall disease incidence.

By mapping study and intervention characteristics, as well as reported outcomes, this review aims to provide an overview of current practices, highlight promising approaches, and identify gaps in the evidence base that warrant further research or policy attention.

The review addresses the following questions:

1. How are diabetes prevention interventions conceptualized, designed, adapted and implemented in community and public health settings, and how are intervention evaluations reported?
2. Which target populations are reached by these interventions?
3. What gaps exist in the current landscape of diabetes prevention initiatives delivered in these settings?

## Methods

### Study design and conceptual framework

A scoping review was conducted to address the broad and exploratory nature of the research questions, providing an overview of the literature while identifying gaps in current knowledge. The review was performed according to the Joanna Briggs Institute (JBI) methodology for scoping reviews and is reported in line with the Preferred Reporting Items for Systematic Reviews and Meta-Analyses Extension for Scoping Reviews (PRISMA-ScR) guidelines [22–24]. The study protocol was previously published [25].

The Population, Concept, and Context (PCC) framework was used to structure the research question and define the selection criteria [26]:

- *Population*: Adults (≥ 18 years), including healthy individuals and those at increased risk of T2DM (e.g., individuals with prediabetes, as defined by WHO and ADA, or metabolic syndrome)
- *Concept*: Lifestyle-based interventions aimed at preventing T2DM, with a focus on nutrition, physical activity, and general health-related behaviors
- *Context*: Interventions delivered in community, municipal, or public health settings (e.g., public health departments, community or municipal health centers, centers for disease control and prevention, primary care facilities, and local health services), in line with WHO definitions [17, 18].

### Search strategy and eligibility criteria

The search strategy was developed based on the PCC framework, in consultation with a librarian, and adapted for each database and platform. Keywords included terms such as “(pre)diabetes,” “prevention program,” and “public health,” among others. An additional file shows the search string in more detail (see Supplementary Material 1).

Studies identified through review articles were included if deemed relevant. Only studies published in English or German were eligible. No restrictions were applied regarding cultural, racial, gender, or geographical context.

Given the limited availability of randomized controlled trials outside high-income countries, this scoping review included a broader range of controlled study designs ((cluster-)randomized controlled trials (RCT), non-randomized controlled studies, and controlled clinical trials) to map intervention practices across diverse settings. Additionally, feasibility studies, pilot studies, case studies, uncontrolled implementation studies, and process evaluations were excluded to ensure comparability of intervention design and evaluation approaches across included studies. Further exclusion criteria comprised studies involving individuals with clinically diagnosed diabetes (gestational, type 1, or type 2), and studies with mixed populations (e.g., T2DM and prediabetes or adults and children).

Studies were excluded if they examined pharmacological interventions, commercial weight loss programs, interventions lasting fewer than four weeks [27], or samples comprising fewer than ten participants. Pharmacological interventions were excluded to isolate lifestyle-based prevention strategies and to enhance comparability across interventions, given that pharmacological approaches differ substantially in their mechanisms, delivery, and implementation context.

Interventions delivered in clinical settings, such as hospitals or specialist clinics focused on individual diagnosis and treatment, were excluded. Primary health care settings were included only when interventions were implemented as part of population-oriented prevention strategies, rather than individualized clinical management.

A systematic search of published and unpublished literature was conducted. Searches were performed on 28 May 2025 and covered studies published from 1 January 2014 to 28 May 2025 to ensure relevance to contemporary public health practice. The search was conducted across six electronic databases: PubMed, Web of Science Core Collection, CINAHL (via EBSCOhost), CENTRAL (via Ovid), and ClinicalTrials.gov. The Cochrane Database of Systematic Reviews (CDSR) was searched to identify relevant primary studies through citation tracking.

Additional sources included manual screening of websites of major public health organizations (WHO, Centers for Disease Control and Prevention (CDC), International Diabetes Federation (IDF), and ADA), a structured Google Scholar search (limited to the first five pages), grey literature searches in ProQuest and OAIster, and reference list screening of included studies.

Additionally, an artificial intelligence (AI)-assisted search using undermind.ai (free version) was conducted, supplementing the original protocol to ensure broader coverage; details of the search strategy used on undermind.ai are provided in Supplementary Material 1.

### Selection of sources of evidence and data charting

All identified records were imported into EndNote 21 (Clarivate Analytics, PA, USA) for duplicate removal and subsequently uploaded to Covidence (Veritas Health Innovation, Melbourne, Australia) for screening. Following an initial pilot screening, titles and abstracts were screened independently by two of three reviewers (DM, CW, JP) using predefined eligibility criteria. Studies deemed potentially relevant were retrieved in full text and assessed independently by two of three reviewers (DM, CW, JP). Reasons for exclusion at the full-text stage were documented. Any disagreements were resolved through discussion and, where necessary, in consultation with a third reviewer. All records identified through additional search approaches (grey literature, organizational websites, and undermind.ai) were subjected to the same screening and eligibility assessment procedures as database-derived records.

Data were charted using a standardized extraction form that was iteratively refined in accordance with the PRISMA-ScR guidelines. Two reviewers independently extracted the data, with cross-checking by a third reviewer to ensure accuracy and consistency. The corresponding authors were contacted when clarification of missing or ambiguous information was required. Extracted data included: (a) study characteristics (authors, year of publication, country, setting, study objectives, and design); (b) population characteristics (recruitment, inclusion and exclusion criteria, age, gender, and sample size); (c) intervention characteristics and contextual factors (content of intervention and comparator, duration, delivery agents, community involvement, and theoretical framework); (d) outcome measures (assessment time points, follow-ups, measurement instruments, and primary and secondary outcomes); and (e) key findings relevant to the research questions.

Consistent with scoping review methodology, no formal critical appraisal of methodological quality was undertaken, as the primary aim was to map the available evidence rather than evaluate study rigor [23].

Data were synthesized using a narrative and descriptive approach. Findings were summarized thematically and presented in both textual and tabular formats.

## Results

### Results of the literature search

**Fig. 1:**
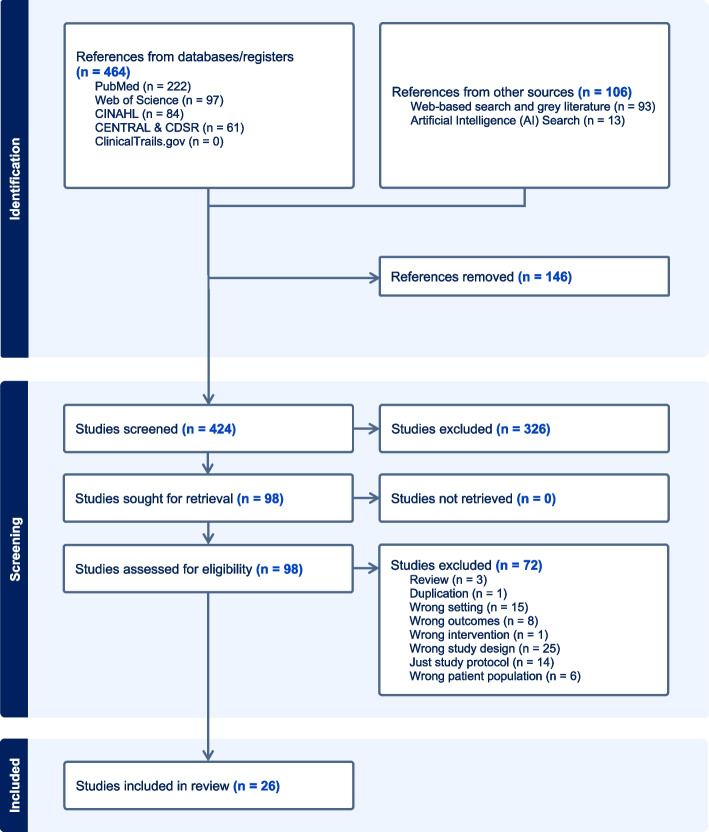
PRISMA-ScR flow chart outlining the inclusion and exclusion of the identified studies (*n* = 26)

In total, the search yielded 464 records (PubMed *n* = 222, Web of Science *n* = 97, CINAHL *n* = 84, CENTRAL & CDSR *n* = 61), while no records were retrieved from ClinicalTrials.gov. A further 106 records were identified through manual searches of reference lists, websites, and AI-based searches. Following deduplication, screening and eligibility assessment, 26 studies were included in the review. The study selection process is illustrated in the PRISMA flow diagram (Fig. 1), and the characteristics of the included studies are summarized in Table 1.

**Table 1:** Characteristics of the included studies (*n* = 26)

First Author, Year, Country [citation]	Population	Sample size/gender	Design	*Name of program*/Intervention	Primary outcome(s)
Setting: Public/Primary Health Care					
Aekplakorn 2019, Thailand [28]	Thai adults aged 30–65 years (y) at risk of developing type 2 diabetes mellitus (T2DM)	*N* = 1903 ♂ Not available (N/A) ♀ N/A	Cluster Randomized controlled trial (RCT)	*Thai Diabetes Prevention Program*: Group-based lifestyle workshops including diabetes education, self-management, peer support, and motivation	Incidence rate T2DM
Alfawaz 2019, Saudi Arabia [29]	Saudi adults aged 20–60 y with prediabetes	*N* = 160 ♂ N/A ♀ N/A	RCT	*Education-based lifestyle intervention:* Workshops and lectures on prediabetes management, nutrition, weight management, and physical activity (PA), supported by educational materials and PA promotion	Diet, fasting blood samples
Davies 2016, United Kingdom [30]	British adults at increased risk of T2DM (high proportion of South Asians)	*N* = 880 ♂ 560 (64%) ♀ 320 (36%)	Cluster RCT	*Group-based structured education program:* One 6-h session on lifestyle, diet, and PA, with annual refresher sessions over 3 years, supported by regular phone contact and pedometer use	Incidence of T2DM, progression to T2DM after 3 years
Duggan 2014, United States [31]	Hispanic adults residing in the Lower Yakima Valley at risk of T2DM	*N* = 430 ♂ 126 (29.4%) ♀ 304 (70.6%)	RCT	*Community health worker–led lifestyle education:* Five weekly 1-h guided sessions on diabetes education, awareness, and self-management	Diet, hemoglobin A1c (HbA1c), PA
Duijzer 2017, Netherlands [32]	Dutch adults aged 40–70 y at risk of T2DM	*N* = 316 ♂ N/A ♀ N/A	RCT	*SLIM iMplementation Experience Region Noord- en Oost-Gelderland (SLIMMER)*: Multicomponent lifestyle intervention including tailored dietary counseling (≈5–6 individual dietitian visits and 1 group session), weekly physiotherapist-led PA sessions, and nurse-led telephone case management for 10 months, followed by a 3-month maintenance program	Fasting insulin
Fischer 2016, United States [33]	English- and Spanish-speaking adults with prediabetes	*N* = 163 ♂ N/A ♀ N/A	RCT	*Diabetes prevention program with text messages (based on National Diabetes Prevention Program (NDPP)):* Digital lifestyle intervention delivering 6 text messages per week (English or Spanish) on diet, PA, motivation, and self-monitoring, with optional phone-based motivational interviewing by a health coach	Body weight
Juul 2016, Denmark [34]	Danish adults at high risk of T2DM	*N* = 127 ♂ 40 (31%) ♀ 87 (69%)	RCT	*Group-based health education program:* Four 2-h sessions over 5 weeks, plus two booster sessions at 1 and 6 months, using dialogue-based learning and reflection	Weight reduction > 5%, total-fat intake < 30% of energy intake, saturated-fat intake < 10% of energy intake, fiber-intake ≥ 15 g/1000 kcal, changes in PA level
Lakka 2023, Finland [35]	Finnish adults at risk of developing T2DM	*N* = 2907 ♂ N/A ♀ N/A	RCT	*Stop Diabetes (StopDia)*: Hybrid lifestyle intervention with 12-month access to the BitHabit web app (habit-based digital support) plus six 2-h face-to-face group sessions delivered by trained health care professionals	Body weight, diet quality, fasting plasma glucose, PA
Muilwijk 2021, India, Pakistan, Sri Lanka, United Kingdom [36]	South Asian adults at high risk of T2DM	*N* = 3684 ♂ 1859 (51%) ♀ 1811 (49%) ♂ Control Group (CG): 894 (48%) ♂ Intervention Group (IG): 965 (52%) ♀ CG: 943 (52%) ♀ IG: 868 (48%)	Cluster RCT	*Family-based culturally adapted lifestyle intervention:* Delivered by community workers with 9 visits (4 individual and 5 group sessions) and 13 follow-up telephone calls, targeting dietary improvement and lifestyle modification to increase PA	Reductions in body weight (aim > 7% reduction), waist circumference (aim > 5 cm reduction), blood pressure, HbA1c at 12 months
Vlaar 2017, Netherlands [37]	South Asian adults living in the Netherlands at risk of T2DM	*N* = 536 ♂ N/A ♀ N/A	RCT	*Study on Lifestyle Intervention and Impaired Glucose Tolerance Maastricht (SLIM)*: Culturally tailored lifestyle intervention with 6–8 individual dietitian sessions over 6 months, followed by 3–4 booster sessions over 18 months, plus one family home session, two group cooking classes, and a 20-week coach-monitored PA program	Diet, PA, social-cognitive determinants
Wani 2020, Saudi Arabia [38]	Predominantly overweight or obese Saudi adults aged ≥ 20 y at risk of T2DM	*N* = 300 ♂ N/A ♀ N/A	RCT	*Intensive Lifestyle Monitoring Program*: Individualized lifestyle counseling with dietitian and physical therapist consultations, supported by ongoing follow-up via phone, email, or SMS, focusing on dietary modification and PA with pedometer-based self-monitoring	Body weight, fasting blood glucose
Yin 2018, China [39]	Chinese women aged 25–65 y with prediabetes	*N* = 184 ♀ 184 (100%)	RCT	*Pathway to Health (PATH)*: lifestyle intervention delivered over 6 months, comprising 12 weekly 60-min large-group education sessions and 10 small-group sessions for goal setting, counseling, and social support, led by trained community health educators, with weekly telephone follow-up, targeting ≥ 5% weight loss, increased PA, and dietary energy reduction	Body weight, diet, fasting glucose, HbA1c, PA
Setting: Public/Primary Health Care + Community-based					
Dunbar 2015, Australia [40]	Australian adults at risk of developing T2DM	*N* = 342 ♂ 129 (37.7%) ♀ 213 (62.3%)	RCT	*Life!*—*Melbourne Diabetes Prevention Study (MDPS):* Lifestyle intervention comprising one 30–45-min individual session and five 90-min group sessions delivered biweekly, with an 8-month booster session, targeting diet, PA, weight loss, and behavior change	Estimated change in the risk of progression to diabetes and cardiovascular disease based on changes in body weight, waist circumferences, glucose measures, blood pressure and lipids
Sampson 2021, United Kingdom [41]	Adults at high risk of T2DM defined by impaired fasting glucose (IFG) or prediabetic-range HbA1c in eastern England	*N* = 1028 ♂ CG: 108 (60.7%) ♂ IG1 258 (60.8%) ♂ IG2: 279 (65.5%) ♀ CG: 70 (39.3%) ♀ IG1 166 (39.2%) ♀ IG2: 147 (34.5%)	RCT (with 3 arms)	*Norfolk Diabetes Prevention Study: Theory-based group lifestyle intervention with s*ix 2-h core sessions over 12 weeks, followed by up to 15 maintenance sessions every 8 weeks including 50-min supervised PA, delivered by health care professionals with optional Diabetes Prevention Mentor support (IG2)	Incidence of T2DM
Yeh 2016, United States [42]	Chinese immigrant adults in New York City with prediabetes (Body mass index (BMI) ≥ 23 kg/m^2^; HbA1c 5.7%–6.4%)	*N* = 60 ♂ N/A ♀ CG: 15 (50%), ♀ IG: 19 (63.3%)	RCT (with 2 arms)	*Culturally and linguistically tailored lifestyle intervention:* Twelve biweekly core sessions and 6 monthly maintenance sessions (1.5–2 h each) covering diet, PA, stress management, and behavior change, delivered in Mandarin or Cantonese	BMI, HbA1c, weight change
Setting: Community-based					
Fianu 2016, La Réunion Island [43]	Adults living in socioeconomically deprived areas in Reunion Island at risk of T2DM	*N* = 439 ♂ N/A ♀ N/A	Cluster controlled trial	*REunion DIAbetes primary prevention (REDIA-prev1)*: Lifestyle program including diet workshops, free PA offers (daily gym access, group walks 3 times per week for 2 h, and periodic hikes and recreational sports), and six support group sessions; participation was free and unrestricted	BMI, body weight, waist circumference
Hays 2016, United States [44]	Economically disadvantaged adults with prediabetes	*N* = 216 ♂ 61 (28.2%) ♀ 155 (71.8%)	RCT	*RAPID/Young Men’s Christian Association Diabetes Prevention Program (YDPP)*: Group-based diabetes prevention program with 16 sessions (60–90 min each) focusing on diet, PA, and behavior change, followed by monthly 60-min maintenance sessions, targeting 7% weight loss and ≥ 150 min/week of home-based moderate PA	Changes in moderate-to-vigorous PA
Lotfaliany 2020, Iran [45]	Adults aged ≥ 20 y without T2DM living in Tehran	*N* = 9204 ♂ N/A ♀ N/A	Cluster controlled trial	*Tehran Lipid and Glucose Study (TLGS):* Multilevel lifestyle intervention comprising individual consultation + 2-h small-group education, community gatherings 2–4 ×/year (1.5–3 h) with health promotion activities, and school-based programs (students: 12 × 45-min sessions/year in grade 1 and 3 sessions/year in grades 2–3; parents: 3 × 60-min sessions/year; teachers/staff: annual 2-day seminars + 45-min classes), supported by newsletters and printed materials	Incidence of T2DM
Manios 2020, Belgium, Bulgaria, Finland, Greece, Hungary, Spain [46]	Parents from vulnerable population groups at risk of T2DM	*N* = 2756 ♂ 926 (33.6%) ♀ 1830 (66.4%)	Cluster RCT	*Feel4Diabetes intervention:* Family- and community-based lifestyle intervention with a universal school-delivered “All Families” component promoting healthy diet and PA, and a “High-Risk Families” component consisting of 1 initial counseling session plus 5 follow-up counseling sessions over 12 months**,** supported by community and environmental physical activity initiatives	Diet
Mayer 2019, United States [47]	Obese adults with prediabetes from underprivileged areas, mostly ethnic minorities (73% Latino, 23% Black or African American), 58% non–English-speaking	*N* = 402 ♂ 59 (15%) ♀ 343 (85%)	RCT	*Community-based peer-led lifestyle intervention:* Eight 90-min workshops delivered in English or Spanish, focusing on diet, PA, stress management, and behavior change through peer support	Percentage weight loss, change in probability of developing diabetes over the next 7.5 years
Odglun 2023, Thailand [48]	Thai Muslim adults at risk for T2DM	*N* = 119 ♂ CG: 21 (35.6%) ♂ IG: 28 (46.7%) ♀ CG: 38 (64.4%) ♀ IG: 32 (53.3%)	Cluster RCT	*Peer- and religion-based lifestyle intervention:* Peer-leader training comprising three group sessions and self-learning via the LINE app, followed by participant education including one 3-h group session, one 20-min home visit, one booster group session, three 20-min small-group booster sessions, and ongoing LINE-based support	Diabetes prevention knowledge, anthropometric weight, BMI, waist circumference, HbA1c and lipid profile
Patel 2017, United States [49]	Gujarati Asian Indian adults in an urban US community	*N* = 70 ♂ N/A ♀ N/A	RCT	*National Diabetes Education Program’s (NDEP) Power to Prevent (P2P)/modified:* Weekly group-based lifestyle intervention with 12 sessions (75 min each), including facilitator-led 20-min group PA in eight sessions, supported by text-message reminders, self-monitoring tools (pedometers), specific, measurable, achievable, realistic, time-based (SMART) goal setting	Body weight, HbA1c, PA
Pengpid 2018 Thailand [50]	Thai temple members aged 35–65 y with prehypertension and/or prediabetes (IFG/blood pressure)	*N* = 443 ♂ CG: 58 (26%) ♂ IG: 56 (25.5%) ♀ CG: 165 (74%) ♀ IG: 164 (74.5%)	Cluster RCT	*Group-based lifestyle counseling*: Six sessions (60–90 min) delivered over 6 months by trained nurses, focusing on diet, PA, goal setting, and diabetes education, with homework and evaluation	Fasting plasma glucose, blood pressure (systolic/diastolic), lipid profiles, BMI, waist circumference
Smith 2019, United Kingdom [51]	Adults at risk of developing T2DM	*N* = 315 ♂ 137 (44%) ♀ 178 (56%)	RCT	*Living Well, Taking Control (LWTC)/Community-based Prevention of Diabetes (Com-PoD)*: Group-based lifestyle intervention with four weekly 2-h sessions, followed by support contacts at 2, 3, 6, 9, and 12 months (group-based or by telephone), plus 5 optional hours of participant-chosen activities	Body weight
Thankappan 2018, India [52]	Indian adults aged 30–60 y at risk of developing T2DM	*N* = 1007 ♂ N/A ♀ N/A	Cluster RCT	*Kerala Diabetes Prevention Program (K-DPP)*: Peer-support lifestyle intervention delivered over 12 months, comprising 15 group sessions (12 led by trained lay peer leaders) plus ongoing peer contact and community activities, targeting PA, healthy diet, weight management, and risk behaviors	Incidence of T2DM
VanName 2016, United States [53]	Inner-city, low-income mostly Hispanic adult women at risk of T2DM	*N* = 130 ♂ N/A ♀ N/A	RCT	*Family Health Center Healthy Lifestyle Intervention (FHCHC ILI)*: Family-centered lifestyle intervention delivered over 14 weeks, including weekly 1-h lifestyle education sessions and trainer-led group exercise 2–3 times per week (1 h each), with parallel play-based PA for children and family participation	Body weight

*Abbreviations*: *BMI* Body mass index, *CG* Control group, *Com-PoD* Community-based Prevention of Diabetes, Combined Prevention of Diabetes study, *FHCHC ILI* Family Health Center Healthy Lifestyle Intervention, *HbA1c* Hemoglobin A1c, *IFG* Impaired fasting glucose, *IG* Intervention group, *K-DPP* Kerala Diabetes Prevention Program, *LWTC* Living Well, Taking Control, *MDPS* Life!—Melbourne Diabetes Prevention Study, *MVPA* Moderate-to-vigorous physical activity, *N/A* Not available, *NDEP* National Diabetes Education Program, *NDPP* National Diabetes Prevention Program, *PA* Physical activity, *PATH* Pathway to Health, *P2P* Power to Prevent, *RCT* Randomized controlled trial, *REDIA-prev1* REunion DIAbetes primary prevention, *SLIM* Study on Lifestyle Intervention and Impaired Glucose Tolerance Maastricht, *SLIMMER* SLIM iMplementation Experience Region Noord- en Oost-Gelderland, *SMART* Specific, measurable, achievable, realistic, time-based (goal setting), *StopDia* Stop Diabetes, *T2DM* Type 2 diabetes mellitus, *TLGS* Tehran Lipid and Glucose Study, *YDPP* Young Men’s Christian Association Diabetes Prevention Program, *y* years

### Characteristics of the included studies

**Fig. 2:**
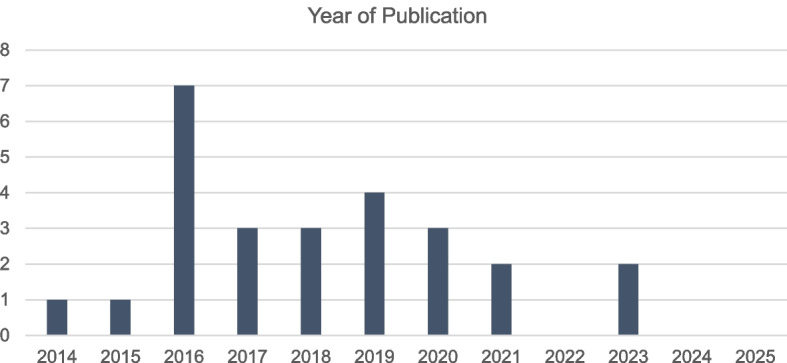
Bar chart showing the publication years of the studies (*n* = 26)

**Fig. 3:**
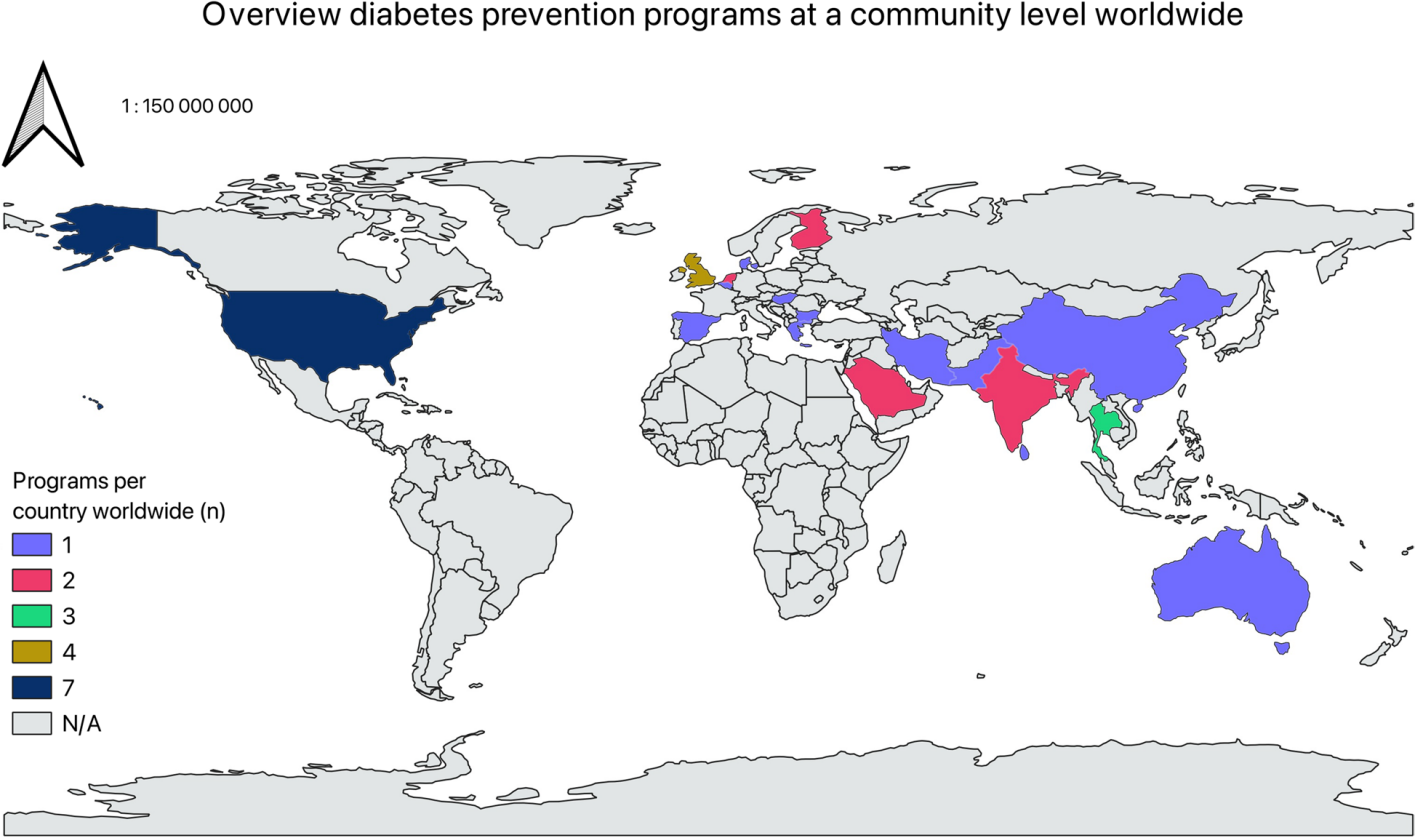
Overview diabetes prevention programs at a community level worldwide (*n* = 26)

The included studies were published between 2014 and 2023, with the highest number of publications in 2016. No eligible studies were published in 2022, 2024, or 2025 (see Fig. 2). Most studies were conducted in the United States (*n* = 7), followed by the United Kingdom (*n* = 4) and Thailand (*n* = 3) (see Table 1, see Fig. 3). Overall, interventions were implemented across 19 countries, with two studies conducted in multiple countries [36, 46]. According to the World Bank Group income classification [54], most studies were conducted in high-income countries (HIC; *n* = 19; 74%), followed by upper-middle-income countries (UMIC; *n* = 5; 19%) and lower-middle-income countries (LMIC; *n* = 2; 7%). No studies have been conducted in low-income countries (LIC).

Seventeen studies (65%) employed an RCT design, seven studies (27%) were cluster-RCTs, and two used non-randomized cluster-controlled trial designs (see Table 1). Among studies conducted in middle-income countries (MIC) (*n* = 7), one used an RCT design [39], while the remaining studies applied cluster-based designs. Among studies conducted in HICs (*n* = 19), most used RCT designs (*n* = 16), with three studies employing alternative designs (cluster-RCTs or controlled trials) [30, 43, 46] (see Table 1). In studies where reasons for the use of cluster-based designs were reported, these included family- or community-level implementation [46], cost considerations [46], infeasibility of individual randomization [28], the emphasis on the community aspect without a link to the design choice [43, 45], and potential contamination between study arms [52].

The study populations comprised adults, with a mean lower age limit of 27.4 years and a median of 25 years. Most studies included both male and female participants; two studies enrolled women only [39, 53]. Sample sizes ranged from 60 [49] to 9204 participants [45], with a mean sample size of 1082 and a median of 372.

One study enrolled adults without increased T2DM risk [45]. Across the remaining studies, elevated T2DM risk was defined via blood-based measures (e.g., IFG, IGT, or hemoglobin A1c (HbA1c)), anthropometric criteria (e.g., body mass index (BMI) or obesity thresholds), or validated diabetes risk scores (e.g., Finnish Diabetes Risk Score (FINDRISC) [55], Australian Type 2 Diabetes Risk Assessment Tool (AUSDRISK) [56] or Indian Diabetes Risk Score (IDRS) [57]). Eight studies specifically recruited participants with prediabetes [29, 33, 39, 41, 42, 44, 47, 50].

Several studies have applied sociodemographic criteria to target specific population groups, including economically disadvantaged communities [42–44, 46, 47, 53] and ethnic, religious or linguistic minority populations within the study setting (e.g., Hispanics [31, 47, 53], South Asians [37, 49, 53] or a Muslim population in Thailand [48]).

Twelve studies (44%) were implemented as community-based interventions in settings such as community centers, religious venues (e.g., mosques, temples), Young Men's Christian Association (YMCA) facilities, community halls, schools, neighborhood facilities, and local charities [44–46, 48–51]. These interventions were often publicly funded and delivered by universities [36, 46–48, 53], non-governmental organizations (NGOs) [44], or a collaboration of these entities [51]. Ten studies (37%) were conducted within public or primary health care settings, including public health departments, primary care practices, dietician practices, and health screening centers. Several studies reported combined delivery formats, with recruitment or screening conducted in public/primary health care-based settings and intervention delivery taking place in community locations or participants’ homes [36, 46]. All cluster trials conducted in MICs described interventions as community-based or delivered in community settings, compared with seven of 19 studies conducted in HICs.

### Intervention characteristics and core components

#### Intervention development

**Fig. 4:**
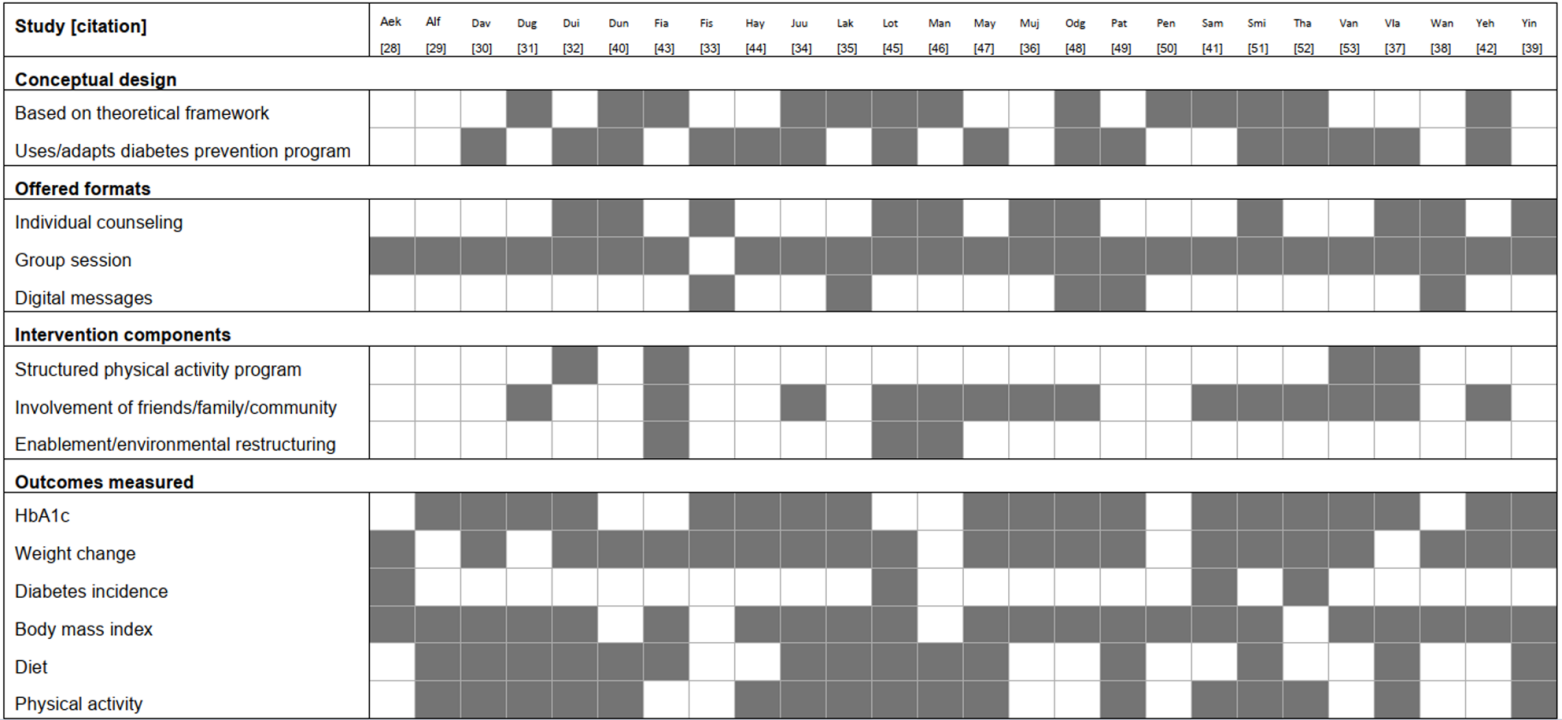
Matrix overview of the characteristics of the intervention (*n* = 26) Abbreviations. HbA1c, hemoglobin A1c. Note. A structured physical activity (PA) program is only defined as such if it encompasses regular, structured offers of PA that amount to at least the weekly recommended amount for healthy living according to World Health Organization (WHO) guidelines; the offer of an open gym for this amount of time does not suffice. Enablement/environmental restructuring describes measures to reshape the daily environment of participants to enable and promote healthy eating and/or PA beyond the intervention offers themselves. Examples include discounts for fruits and vegetables in the supermarket, labelling of school snacks, subsidies for local gyms, local public sports competitions, developing sports facilities etc. For studies with more than one intervention arm or intervention differences between clusters, the more extensive offer is marked here

Fifteen studies reported adapting and implementing existing DPP, most frequently the US NDPP or National Diabetes Education Program (NDEP) (*n* = 5) [12] (see Table 2, see Fig. 4).

**Table 2:** Theoretical backgrounds and model programs of included intervention studies

First Author, Year [citation]	Theoretical background	Model program
Davies 2016 [30]	Not specified	Tailored Diabetes Education and Self-Management for Ongoing and Newly Diagnosed (DESMOND) structured-education program integrated into a prevention context; incorporation of the PREPARE program
Duggan, 2014 [31]	Social Cognitive Theory (Bandura)	Not specified
Duijzer 2017 [32]	Not specified	SLIM (Study on Lifestyle Intervention and Impaired Glucose Tolerance Maastricht), translated into SLIMMER (SLIM iMplementation Experience Region Noord- en Oost-Gelderland)
Dunbar 2015 [40]	Health Action Process Approach (HAPA)	Adapted from Finnish Diabetes Prevention Study (FIN-DPS); Good Ageing in Lahti (GOAL); Australian Greater Green Triangle (GGT) Diabetes Prevention Project; resulted in Life!
Fianu 2016 [43]	Whitehead’s categories of interventions for health action	Not specified
Fischer 2016 [33]	Not specified	National Diabetes Prevention Program (NDPP) adapted to include text messaging
Hays 2016 [44]	Not specified	Group-based adaptation of the National Diabetes Education Program (NDEP) in collaboration with the Young Men’s Christian Association (YMCA)
Juul 2016 [34]	Transformative Learning Theory (Mezirow); Health Literacy Theory	FIN-DPS; GOAL
Lakka 2023 [35]	Self-Determination Theory; Self-Regulation Theory; Habit Formation Theory	Not specified
Lotfaliany 2020 [45]	American Heart Association guidelines	Adaptation of the North Karelia Project informed by Knowledge, Attitude and Practice (KAP) studies
Manios 2020 [46]	PRECEDE–PROCEED model; Health Action Process Approach (HAPA); specific, measurable, attainable, realistic and timely (SMART) goals	Not specified
Mayer 2019 [47]	Not specified	Adaptation of American Association of Diabetes Education Standards; Stanford Chronic Disease Self-Management Program
Odglun 2023 [48]	Health Belief Model; Social Support framework	NDPP and FIN-DPS adapted using the Intervention Mapping approach
Patel 2017 [49]	Not specified	NDEP Power to Prevent (P2P), adapted for Asian Indians
Pengpid 2018 [50]	HAPA; Self-regulation theory	Not specified
Sampson 2021 [41]	Behavioral theory-based approach including social support and self-management; peer mentors in the Intensive Diabetes Management (INT-DM) arm, Empowerment Theory, HAPA	Not specified
Smith 2019 [51]	National Institute for Health and Care Excellence (NICE) recommendations for diabetes prevention	Adaptation of diabetes management programs developed by Westbank (Exeter) and Health Exchange (Birmingham), comprising the prototype "Living Well, Taking Control" (LWTC) program
Thankappan 2018 [52]	HAPA; socio-behavioral lifestyle intervention model; Intervention Mapping	Adaptation of GOAL and GGT
VanName 2016 [53]	Not specified	Modified National Institute of Diabetes and Digestive and Kidney Diseases Diabetes Prevention Program
Vlaar 2017 [37]	Not specified	Adaptation of SLIM
Yeh 2016 [42]	Community-based participatory research approach	Adaptation of NDPP (based on feedback from 3 focus groups)

Studies that did not report either a theoretical framework or a model program were excluded from this table

*Abbreviations*: *DPP* Diabetes Prevention Program, *DESMOND* Diabetes Education and Self-Management for Ongoing and Newly Diagnosed, *FIN-DPS* Finnish Diabetes Prevention Study, *GGT* Greater Green Triangle, *GOAL* Good Ageing in Lahti, *HAPA* Health Action Process Approach, *INT-DM* Intensive Diabetes Management, *KAP* Knowledge, Attitude, and Practice, *LWTC* Living Well, Taking Control, *NDEP* National Diabetes Education Program, *NICE* National Institute for Health and Care Excellence, *P2P* Power to Prevent, *SLIM* Study on Lifestyle Intervention and Impaired Glucose Tolerance Maastricht, *SLIMMER* SLIM iMplementation Experience Region Noord- en Oost-Gelderland, *SMART* Specific, measurable, attainable, realistic, time-based; *NDPP* National Diabetes Prevention Program; YMCA, Young Men’s Christian Association

Ten studies reported the use of theoretical or conceptual frameworks based on behavioral change theories such as the Health Action Process Approach (HAPA) (*n* = 4) [58], Self-regulation theory [e.g. 59, 60] and the Intervention Mapping Approach [61] to either develop their own interventions or adapt a program (see Table 2, see Fig. 4).

#### Intervention timeframe

The duration of interventions ranged from 3 months [e.g. 29, 31] to 36 months including a maintenance period [30]. The mean duration was 12.43 months, with a median of 12 months. Eight studies (≈30%) reported intervention durations of ≤ 6 months, eleven studies (≈42%) reported durations of 6–12 months, and six studies reported durations of ≥ 12 months; one study did not clearly report duration. Twelve studies (~ 46.1%) either had a designated maintenance period or faded the sessions out as part of the intervention.

#### Intervention format and content

All included interventions included educational components related to T2DM prevention, including risk factors, self-management, healthy diet, and physical activity. Most studies (*n* = 25) included group-based sessions or workshops, while some studies also incorporated individual counseling sessions, particularly in relation to dietary guidance [32, 38]. Within group-based sessions, behavioral components such as goal setting, self-monitoring, and problem-solving were frequently described [e.g. 28, 31, 40, 41, 62], although explicit reporting of behavior change techniques [62] was limited (see Fig. 4).

#### Intervention activities

Six programs included practical activities, such as grocery shopping or cooking classes [37, 43, 47, 49, 51, 53]. Most studies (*n* = 23) reported physical activity components, either as recommendations or as part of the intervention. Four studies included structured, supervised physical activity sessions aligned with WHO-recommended activity levels [32, 37, 53, 63], while four reported limited supervision, with participants primarily engaging in self-directed activity [41, 42, 47, 49]. Several studies reported digital components such as text messaging or mobile applications [33, 35, 38, 48, 49] (see Fig. 4).

#### Community involvement

Community involvement was defined in line with WHO guidance as the inclusion of individuals, families, or community stakeholders in intervention delivery, activities, or supportive structures, beyond participation in standard intervention sessions [64]. Seventeen studies described components aimed at promoting social support and healthy routines, including the involvement of family members or peers, community events, or activities addressing environmental barriers to healthy behaviors [e.g. 31, 43, 45, 46, 48, 53].

### Outcomes reported across diabetes prevention interventions

All but one study [29] clearly specified primary outcomes, usually alongside multiple secondary outcomes. The most frequently reported outcomes included T2DM incidence or risk, body weight, physical activity, and dietary behavior, which were assessed either individually or in combination (see Fig. 4).

Most studies reported collecting blood-based measures, including HbA1c and other metabolic markers (e.g., fasting glucose, insulin, high-density lipoprotein, low-density lipoprotein, and triglycerides); two studies did not report blood-based outcomes [43, 46]. Anthropometric measures, such as BMI, body weight, or waist circumference, were reported in all but two studies [31, 46]. Dietary outcomes were assessed exclusively using self-report methods, most commonly full [31, 32, 34, 35, 40, 45, 46] or abbreviated food frequency questionnaires (FFQs) [30, 37, 41, 47, 52, 65]; two studies used dietary recall methods [29, 43]. Physical activity was predominantly assessed via self-report instruments, most frequently the International Physical Activity Questionnaire (IPAQ) [30, 34], or modified versions thereof [31, 36, 39, 41, 46, 51, 52]. However, a few studies have also reported objective methods, such as an accelerometer [44, 51], a pedometer [30] and a walking test [32].

## Discussion

This scoping review aimed to map lifestyle-based diabetes prevention interventions delivered in public health, municipal, and community settings. From a broad initial evidence base, 26 studies were identified that met the inclusion criteria. The findings indicate that community- and public-health-based T2DM prevention initiatives are predominantly group-based, education-focused, and largely adapted from established model programs, with variation in study design and intervention duration, and limited reporting of digital components and theoretical frameworks. While most interventions targeted similar outcomes, considerable variation was observed in how programs were implemented, evaluated, and embedded within local contexts.

A key finding was the widespread adaptation of established prevention programs. While this reflects the translation of existing evidence into applied settings, the processes by which adaptations were conducted were not consistently reported. In many cases, it remained unclear how core components of the original interventions were modified, retained, or operationalized. Similarly, reporting of theoretical frameworks was limited. Although behavioral components, such as goal setting, self-monitoring, and problem-solving, were frequently described, they were often not explicitly linked to underlying mechanisms. This pattern is consistent with previous observations in diabetes prevention research and highlights a gap in the reporting of theoretical and mechanistic foundations [6–9].

The definition and reporting of “community-based” approaches varied considerably across studies and represent a central finding of this review. Across the included studies, “community-based” encompassed a broad range of contexts, including delivery in non-clinical venues such as community centers, schools, religious institutions, or local organizations, as well as interventions linked to primary health care or public health services. In some cases, the term appeared to reflect the location of delivery, while in others it referred more broadly to elements of community involvement. Community involvement itself was reported in different forms, including group-based formats, the involvement of family members or peers, or activities linked to local environments. However, these components were often described only briefly and did not consistently specify the level, depth, or processes of engagement. As a result, it remained unclear to what extent interventions were embedded within community structures beyond their physical setting. This variability in both the definition of “community-based” and the reporting of community involvement limited comparability across studies.

Interventions were predominantly delivered in group-based, education-focused formats, which facilitate delivery at scale and are consistent with the structure of established diabetes prevention programs. The use of digital components was limited in the included studies, whereas other reviews in different settings have reported a greater integration of digital formats [9]. Intervention duration also differed compared with Aziz et al. [9], with a higher proportion of shorter interventions (≤ 6 months) and a lower proportion of longer interventions (≥ 12 months) observed. However, these differences should be interpreted with caution, as they may reflect variations in study selection, design, and reporting rather than consistent changes in intervention practice.

The included studies were published between 2014 and 2023, with no eligible studies identified for 2024 or 2025. This may reflect publication and indexing delays rather than an absence of ongoing research activity. Most studies were conducted in high-income countries, particularly in the United States, consistent with the concentration of diabetes prevention research in these settings [66]. In contrast, relatively few studies were conducted in middle-income countries, and no studies were identified from low-income settings.

A range of study designs was observed across the included studies. Individually randomized controlled trials were most frequently used, while cluster-randomized designs were also commonly applied, particularly in studies conducted in middle-income countries [67, 68]. The use of cluster-based designs aligns with the delivery of interventions in community and public health settings, where allocation often occurs at the group or site level.

### Strengths and limitations

The strength of this review lies in its potential to inform the development of public health programs for diabetes prevention. By identifying and examining common T2DM prevention approaches, it complements prior reviews that have focused primarily on national programs or clinical settings and addresses a gap in the literature on community-level and public health department-based interventions.

This review has several limitations. While effectiveness outcomes are reported across studies, the heterogeneity of designs, outcomes and reporting precluded a meaningful synthesis of intervention effectiveness. A scoping approach was therefore chosen to map current practices and identify gaps for future systematic reviews. The restriction to controlled study designs may have excluded relevant evidence from feasibility studies, pilot studies, and implementation research, which could provide additional insights into real-world delivery; however, this decision was made to enhance comparability across interventions. As such, the findings reflect a defined subset of the available evidence base.

In addition, pharmacological interventions were excluded, although they are recommended in some guidelines for high-risk populations, to maintain a focus on lifestyle-based approaches and ensure comparability within community and public health delivery settings.

Only studies published in English or German were included, and relevant evidence published in other languages may have been missed. Differences in the understanding of “community-based settings” and public health systems across countries in general should also be considered when interpreting the results.

## Conclusion

This scoping review provides a structured overview of controlled lifestyle interventions for diabetes prevention delivered in community, municipal, and public health-related settings. The included studies demonstrate considerable variation in intervention design, delivery, and reporting. Interventions were commonly based on established program models and delivered in group-based, education-focused formats, while the use of digital components was reported less frequently.

A key finding of this review is the variability in how “community-based” approaches are defined and described. The term encompassed a wide range of settings and levels of community involvement, which were not consistently specified across studies. This heterogeneity, together with limited reporting of theoretical foundations and adaptation processes, restricts comparability and complicates the synthesis of intervention characteristics.

The evidence base remains largely concentrated in high-income countries, with comparatively few studies from middle-income settings and no representation from low-income contexts. Future research may benefit from clearer definitions of community-based delivery, more consistent reporting of intervention components and theoretical approaches, and broader inclusion of diverse study designs and settings.

As this review did not assess study quality or effectiveness, it does not permit conclusions regarding the relative effectiveness of different intervention models. Instead, it maps current practices and identifies gaps in the reporting and implementation of diabetes prevention interventions in community and public health contexts.

## Supplementary Information

Supplementary Material 1.

## Data Availability

The datasets supporting the conclusions of this article are included within the article and its additional file.

## Abbreviations

ADA: American Diabetes Association
AI: Artificial Intelligence
AUSDRISK: Australian Type 2 Diabetes Risk Assessment Tool
BMI: Body mass index
CDC: Centers for Disease Control and Prevention
CCT: Cluster-controlled trial
CENTRAL: Cochrane Central Register of Controlled Trials
CDSR: Cochrane Database of Systematic Reviews
CINAHL: Cumulative Index to Nursing and Allied Health Literature
COVID-19: Coronavirus Disease 2019
DPP: Diabetes Prevention Program
FFQ: Food frequency questionnaire
FINDRISC: Finnish Diabetes Risk Score
FIN-DPS: Finnish Diabetes Prevention Study
HAPA: Health Action Process Approach
HbA1c: Hemoglobin A1c
HIC: High-income country
IDF: International Diabetes Federation
IDRS: Indian Diabetes Risk Score
IFG: Impaired fasting glucose
IGT: Impaired glucose tolerance
IPAQ: International Physical Activity Questionnaire
JBI: Joanna Briggs Institute
LIC: Low-income country
LMIC: Lower-middle-income country
MIC: Middle-income country
NDEP: National Diabetes Education Program
NDPP: National Diabetes Prevention Program
NGO: Non-Governmental Organization
NHS DPP: National Health Service Diabetes Prevention Programme
PCC: Population, Concept and Context Framework
PRISMA-ScR: Preferred Reporting Items for Systematic Reviews and Meta-Analyses – Scoping Review extension
RCT: Randomized controlled trial
T2DM: Type 2 diabetes mellitus
UMIC: Upper-middle-income country
WHO: World Health Organization
YMCA: Young Men’s Christian Association
