# Supplementary material for: Type 2 diabetes prevention programs at the community and municipal level: a scoping review of controlled intervention studies
Source: BMC Public Health. 2026 Jul 1;26:2127. doi: 10.1186/s12889-026-28319-8 (PMC13362028; doi:10.1186/s12889-026-28319-8)
Supplement: Supplementary file 1 — Supplementary Material 1. [file 12889_2026_28319_MOESM1_ESM.docx]

Additional file 1

Search strategy details for four databases and AI search

| 1. PubMed |
| --- |
| (("Prediabetic State"[MeSH Terms] OR "Prediabetes"[Title/Abstract] OR "Prediabetic"[Title/Abstract] OR "Pre diabetes"[Title/Abstract] OR "Pre diabetic"[Title/Abstract]) NOT ("Child"[MeSH Terms] OR "Infant"[MeSH Terms])) AND ("Protective Factors"[MeSH Terms] OR "protective factor*"[Title/Abstract] OR "Healthy Lifestyle"[MeSH Terms] OR "Life Style modification"[Title/Abstract:~2] OR "Life Style modifications"[Title/Abstract:~2] OR "Lifestyle modification"[Title/Abstract:~2] OR "Lifestyle modifications"[Title/Abstract:~2] OR "life style change*"[Title/Abstract] OR "lifestyle change*"[Title/Abstract] OR "Secondary Prevention"[MeSH Terms] OR "Preventive Medicine"[MeSH Terms] OR "Preventive Health Services"[MeSH Terms] OR "Preventive Health Service"[Title/Abstract:~2] OR "prevention*"[Title/Abstract] OR "preventing program"[Title/Abstract:~2] OR "preventing programs"[Title/Abstract:~2] OR "preventive program"[Title/Abstract:~2] OR "preventive programs"[Title/Abstract:~2] OR "preventing programme"[Title/Abstract:~2] OR "preventing programmes"[Title/Abstract:~2] OR "preventive programme"[Title/Abstract:~2] OR "preventive programmes"[Title/Abstract:~2] OR "preventive strateg*"[Title/Abstract] OR "preventing strateg*"[Title/Abstract]) AND ("Public Health"[MeSH Terms:noexp] OR "Public Health Administration"[MeSH Terms] OR "Health Planning Organizations"[MeSH Terms] OR "Public Health Practice"[MeSH Terms:noexp] OR "administrative personnel"[MeSH Terms:noexp] OR "health department*"[Title/Abstract] OR "department of health"[Title/Abstract] OR "Global Health"[MeSH Terms] OR "Global Health"[Title/Abstract] OR "Community Health Services"[MeSH Terms] OR "community health service*"[Title/Abstract] OR "community based health promotion*"[Title/Abstract] OR "centers for disease control and prevention, u s"[MeSH Terms] OR "Centers for disease control and prevention"[Title/Abstract] OR "Center for disease control and prevention"[Title/Abstract] OR "Centres for disease control and prevention"[Title/Abstract] OR "Centre for disease control and prevention"[Title/Abstract] OR "Community Health Centers"[MeSH Terms] OR "community health center*"[Title/Abstract] OR "community health centre*"[Title/Abstract] OR "municipal health center*"[Title/Abstract] OR "municipal health centre*"[Title/Abstract]) AND ("english"[Language] OR "german"[Language]) AND 2014/01/01:3000/12/31[Date - Publication] |
| 1. Web of Science Core Collection (exact Search) |
| TS=((Prediabet* OR "Pre diabet*") NOT (child* OR infant* OR kid OR kids)) AND TS=("Protective Factor*" OR "Healthy Lifestyle" OR (("Life Style" OR Lifestyle) NEAR/2 (modification$ OR change$)) OR ((Preventive OR Preventing) NEAR/2 (Medicine OR Program$ OR strateg* OR "Health Service$")) OR Prevention$) AND TS=((health NEAR/1 department$) OR "Global Health" OR "Community Health Service$" OR "Community based Health promotion$" OR "Centers for disease control and prevention" OR "Center for disease control and prevention" OR "Centres for disease control and prevention" OR "Centre for disease control and prevention" OR "Community Health Center$" OR "Community Health Centre$" OR "Municipal Health Center$" OR "Municipal Health Centre$") AND LA=(English OR German) AND PY=(2014-2030) |
| 1. CINAHL (Ebsco-Host) |
| (((MH "Prediabetic State") OR TI ( Prediabet* OR "Pre-Diabet*") OR AB ( Prediabet* OR "Pre-Diabet*")) NOT (MH "Child+")) AND (((MH "Health Behavior") OR (MH "Life Style Changes") OR (MH "Preventive Health Care")) OR TI ("Protective Factor*" OR "Healthy Lifestyle" OR (("Life Style" OR Lifestyle) N2 (modification* OR change*)) OR ((Preventive OR Preventing) N2 (Medicine OR Program* OR strateg* OR "Health Service*")) OR Prevention*) OR AB ("Protective Factor*" OR "Healthy Lifestyle" OR (("Life Style" OR Lifestyle) N2 (modification* OR change*)) OR ((Preventive OR Preventing) N2 (Medicine OR Program* OR strateg* OR "Health Service*")) OR Prevention*)) AND ((MH "Centers for Disease Control and Prevention (U.S.)") OR (MH "Public Health") OR (MH "Public Health Administration") OR (MH "Public Health Infrastructure") OR (MH "Community Health Centers") OR (MH "Health Facility Departments") OR (MH "Community Health Services") OR TI ((health N1 department*) OR "Global Health" OR "Community Health Service*" OR "Community based Health promotion*" OR "Centers for disease control and prevention" OR "Center for disease control and prevention" OR "Centres for disease control and prevention" OR "Centre for disease control and prevention" OR "Community Health Center*" OR "Community Health Centre*" OR "Municipal Health Center*" OR "Municipal Health Centre*") OR AB ((health N1 department*) OR "Global Health" OR "Community Health Service*" OR "Community based Health promotion*" OR "Centers for disease control and prevention" OR "Center for disease control and prevention" OR "Centres for disease control and prevention" OR "Centre for disease control and prevention" OR "Community Health Center*" OR "Community Health Centre*" OR "Municipal Health Center*" OR "Municipal Health Centre*")) AND ((ZL "german") OR (ZL "english")) AND (PY 2014-2030) |
| 1. CENTRAL & CDSR (Cochrane OVID) |
| ((Prediabetic State.sh,kw. or (Prediabetes or Prediabetic or Pre-Diabetes or Pre-Diabetic).ti,ab.) not (child or Child, Preschool).sh,kw.) and ((Protective Factors or Healthy Lifestyle or Secondary Prevention or Preventive Medicine or Preventive Health Services or Early Intervention, Educational or Primary Prevention or Early Medical Intervention or Health Promotion).sh,kw. or (Protective Factor* or Healthy Lifestyle or ((Life Style or Lifestyle) adj2 (modification* or change*)) or ((Preventive OR Preventing) adj2 (Medicine or Program* or Strateg* OR Health Service*)) OR Prevention*).ti,ab.) and ((Public Health or Public Health Administration or Health Planning Organizations or Health Care Coalitions or Health Planning Councils or Health Systems Agencies or Public Health Practice or Administrative personnel or Community Health Services or Global Health or "Centers for Disease Control and Prevention, U.S." or Community Health Centers).sh,kw. or ((health adj1 department*) or Global Health or Community Health Service* or Community based Health promotion* or Centers for disease control and prevention or Center for disease control and prevention or Centres for disease control and prevention or Centre for disease control and prevention or Community Health Center* or Community Health Centre* or Municipal Health Center* or Municipal Health Centre*).ti,ab.) and (english or german).lg. and 2014:2030.(sa_year) |
| AI search via undermind.ai |
| The query specified study design (randomized or cluster-controlled trials), population (healthy adults or adults at increased risk), intervention characteristics (lifestyle-focused, non-pharmacological, with at least one non-medication arm), and delivery context (public health, community). <https://app.undermind.ai/report/17459f3f5840517c6417c819c6fe49b60ea5f16bd76cceebe236d342bec7c295> |
